# Terminological subset of ICNP^®^ for people with chronic kidney disease on hemodialysis

**DOI:** 10.1590/1980-220X-REEUSP-2024-0076en

**Published:** 2024-12-20

**Authors:** Juliana Otaciana dos Santos, Sílvia Maria de Sá Basílio Lins, Richardson Augusto Rosendo da Silva, Harlon França de Menezes, Halene Cristina Dias de Armada e Silva, Joyce Martins Arimatea Branco Tavares

**Affiliations:** 1 Universidade Federal Fluminense, Escola de Enfermagem Aurora de Afonso Costa, Niterói, RJ, Brazil.; 2 Universidade do Estado do Rio de Janeiro, Faculdade de Enfermagem, Departamento de Fundamentos de Enfermagem, Rio de Janeiro, RJ, Brazil.; 3 Universidade Federal do Rio Grande do Norte, Departamento de Enfermagem, Natal, RN, Brazil.; 4 Universidade do Estado do Rio de Janeiro, Faculdade de Enfermagem, Rio de Janeiro, RJ, Brazil.

**Keywords:** Renal Dialysis, Nursing, Renal Insufficiency, Chronic, Standardized Nursing Terminology, Diálisis Renal, Enfermería, Insuficiencia Renal Crónica, Terminología Normalizada de Enfermería, Diálise Renal, Enfermagem, Insuficiência Renal Crônica, Terminologia Padronizada em Enfermagem

## Abstract

**Objectives:**

To construct and validate a terminological subset of the International Classification of Nursing Practice (ICNP^®^) for people with chronic kidney disease on hemodialysis.

**Method:**

Methodological study developed in accordance with the recommendations of the International Council of Nurses (ICN) and the Brazilian method, in the following stages: construction of ND/NO and NI statements of the ICNP^®^ for nursing practice for people with chronic kidney disease on hemodialysis, based on previously constructed specialized terminology and in accordance with Wanda Horta’s Basic Human Needs Theory; and content validation of the statements by focus groups with specialist nurses. The Content Validity Index was used and statements ≥ 0.80 were validated.

**Results:**

82 diagnoses, 130 outcomes and 556 nursing interventions were constructed. After validation, most of the diagnoses (74.5%), outcomes (72.9%) and nursing interventions (65.8%) were classified under psychobiological needs.

**Conclusion:**

A subset with a predominance of statements related to psychobiological needs was constructed and validated, standing out for being the first directed at the care of people with chronic kidney conditions undergoing hemodialysis treatment.

## RESUMO

**Objetivos:**

Construir e validar um subconjunto terminológico da Classificação Internacional da Prática de Enfermagem (CIPE^®^) para pessoas com doença renal crônica em hemodiálise.

**Método:**

Estudo metodológico desenvolvido conforme as recomendações do Conselho Internacional de Enfermeiros (CIE) e do método brasileiro, nas etapas: construção de enunciados de diagnósticos, resultados e intervenções de enfermagem da CIPE^®^ para prática de enfermagem a pessoa com doença renal crônica em hemodiálise, com base em terminologia especializada construída previamente e de acordo com a Teoria das Necessidades Humanas Básicas, de Wanda Horta; e, validação de conteúdo dos enunciados por grupos focais com enfermeiros especialistas. Foi utilizado o Índice de Validade de Conteúdo, sendo validados os enunciados ≥ 0,80.

**Resultados:**

Foram construídos 82 diagnósticos, 130 resultados e 556 intervenções de enfermagem. Após a validação, a maioria dos diagnósticos (74,5%), resultados (72,9%) e intervenções de enfermagem (65,8%) foram classificados nas necessidades psicobiológicas.

**Conclusão:**

Foi construído e validado um subconjunto com predomínio de enunciados relacionados às necessidades psicobiológicas, destacando-se por ser o primeiro direcionado ao cuidado de pessoas em condições renais crônicas em tratamento hemodialítico.

## RESUMEM

**Objetivos:**

Construir y validar un subconjunto terminológico de la Clasificación Internacional de la Práctica de Enfermería (CIPE^®^) para personas con enfermedades renales crónicas en hemodiálisis.

**Método:**

Estudio metodológico desarrollado conforme a las recomendaciones del Consejo Internacional de Enfermerios (CIE) y del método brasileño, en las etapas: construcción de enunciados de diagnósticos, resultados e intervenciones de enfermagem da CIPE^®^ para práctica de enfermagem a pessoa com doença renal crônica em hemodiálise., com base em terminología especializada construida previamente y de acuerdo con la Teoria das Necessidades Humanas Básicas, de Wanda Horta; e, validação de conteúdo dos enunciados por grupos focales com enfermeiros especialistas. Foi utilizado o Índice de Validade de Conteúdo, sendo validados o enunciados ≥ 0,80.

**Resultados:**

Foro construido 82 diagnósticos, 130 resultados y 556 intervenciones de enfermagem. Después de la validación, la mayoría de los diagnósticos (74,5%), los resultados (72,9%) y las intervenciones de enfermagem (65,8%) fueron clasificados según las necesidades psicobiológicas.

**Conclusión:**

Foi construido y validado um subconjunto con predominio de enunciados relacionados con necesidades psicobiológicas, destacando-se por ser el primero dirigido al cuidado de personas en condiciones crónicas crónicas en tratamiento hemodialítico.

## INTRODUCTION

Chronic kidney disease (CKD) is widely regarded as a main public health problem worldwide due to its high morbidity and mortality^(1)^. Current estimates indicate that approximately 850 million people have chronic kidney disease worldwide^(2)^. The Dialysis Census of the Brazilian Society of Nephrology (SBN) estimates that there are more than 1,538,310 people with chronic kidney disease in Brazil, of whom 157,354 are on dialysis, 96.1% on hemodialysis, with an incidence and prevalence rate of 251 and 771 per million of the population, respectively^(3)^.

Hemodialysis (HD) is one of the most widely used renal replacement therapies for people with stage 5 CKD, which aims to maintain life. However, the therapeutic process has negative repercussions on the individual’s life, leading to various biopsychosocial changes^(1, 4)^. In this scenario, the nurse plays a fundamental role in identifying the real and potential affected needs of the client, in order to plan the care provided and draw up an individualized care plan, through the application of the Nursing Process^(5, 6)^.

Based on this perspective, Wanda Horta’s Basic Human Needs Theory (BHN) was adopted in this study, as it is understood that people with chronic kidney disease and on hemodialysis can experience constant imbalances during treatment and that nursing, as an integral part of the healthcare team, through its assistance, contributes to their balance in meeting their basic needs^(7)^.

For organized care, it is essential to use Standardized Language Systems (SLS), being the main among them the International Classification for Nursing Practice (ICNP^®^)^(8)^. In order to maximize the adoption of a unified language accessible to nurses, the International Council of Nurses (ICN) encouraged the construction of terminological subsets of the ICNP^®^, which consist of a set of statements of nursing diagnoses/outcomes (ND/NO) and nursing interventions (NI) appropriate for a particular area of care^(9)^.

The terminological subsets need content validation, an essential step in analyzing their effectiveness and operationality and consolidating the terminology among nurses. Their use will favor the implementation of the nursing process, the construction of computerized health systems, as well as generating data to be used to support and improve clinical practice, the decision-making process, research and professional training^(5, 8, 9, 10, 11)^.

A growing number of terminological subsets have been developed worldwide. In an extensive search of the literature in national and international databases, two publications of subsets in the field of nephrology were found, but these dealt with end-stage CKD and conservative treatment^(12,13)^. This justifies the novelty of this study, since there is no terminological subset aimed at the care of people with chronic kidney disease undergoing hemodialysis.

Against this backdrop, this study aims to build and validate a terminological subset of the ICNP^®^ for people with chronic kidney disease on hemodialysis.

## METHOD

### Study Design

Methodological study, carried out in two stages, the first between September 2020 and August 2022 and the second between May and June 2024. The research was carried out in accordance with the recommendations of the CIE and the Brazilian method for the construction of terminological subsets of the ICNP^®(9, 10, 14)^, which are: 1) the construction of statements of ND/NO and NI of the ICNP^®^ for nursing practice for people with chronic kidney disease on hemodialysis, based on previously constructed specialized terminology and in accordance with Wanda Horta’s Basic Human Needs Theory; and, 2) content validation of the statements by specialist nurses.

### Selection of Experts

In the content validation phase, which took place between May and June 2024, nurses selected using the following inclusion criteria acted as experts: specialists in nephrology or experience in hemodialysis of at least 5 years or nursing professors in nephrology or with published research on ICNP^®^. Thus, nurses from two research groups from public higher education institutions in Brazil participated as experts in the study, who had experience in the construction of terminological subsets of the ICNP^®^. These groups are registered in the Directory of Research Groups in Brazil of the National Council for Scientific and Technological Development (CNPq). In addition to referrals by other research participants, using the snowball technique, in which a participant refers another participant who meets the inclusion criteria.

To define the sample, the study used the following formula: n = Z2 1-α/2. p.(1-p) /e2, where “Z2 1-α/2” = confidence level; “p” = expected proportion of experts; and “e” = acceptable proportion difference in relation to what would be expected. A confidence level of 95% (Z2 1-α/2 = 1.96), an expected proportion of 85% of experts and a sampling error of 15% were considered, resulting in an ideal sample of 22 experts.

### Study Protocol

For the first stage, the empirical basis used was the specialized nursing terminology for chronic kidney patients on hemodialysis, an excerpt from the master’s thesis of the Postgraduate Professional Master’s Program in Nursing Care at the Fluminense Federal University^(15)^. From the specialized nursing terminology constructed, 1,257 terms were obtained referring to the hemodialysis treatment of chronic kidney disease, which made it possible to construct the statements of diagnoses/results (ND/NO) and nursing interventions (NI)^(16)^.

The nursing diagnoses, outcomes and interventions were constructed in line with the recommendations of the ICN and using four empirical bases: the terminology for nursing practice with chronic kidney disease patients on hemodialysis^(15)^, the Seven Axes Model of the ICNP^®(8)^, the ISO 18.104:2023 standard^(17)^ and Wanda Horta’s Basic Human Needs Theory^(7)^.

The recommendations of Vidigal’s study were adopted for the construction of the nursing interventions, and nursing interventions were constructed based on the nurse’s clinical practice and those already validated in other terminological subsets of the ICNP^®(18, 19)^.

As a result, the ND/NRs and NIs were manually submitted to the cross-mapping process, resulting in a list of nursing diagnoses, outcomes and interventions that were and were not included in ICNP^®^ version 2019/2020. The non-constant nursing diagnoses, outcomes and interventions were analyzed independently by two researchers for their degree of equivalence and cardinality, as recommended by ISO/TR 12.300/2016^(20)^.

Conceptual and operational definitions were then drawn up for each nursing diagnosis/outcome, using the ICNP^®^ version 2019/2020, dictionaries of technical health terms and the Portuguese language, scientific articles and other validated ICNP^®^ terminology subsets. The ND/NO and NI were distributed into the following levels of Wanda de Aguiar Horta’s Basic Human Needs (BHN): psychobiological, psychosocial and psychospiritual needs, and subjected to content validation.

In the content validation stage, the data was collected using focus groups via videoconferences on the Google Meet^®^ digital platform. An invitation letter, the Informed Consent Form and the two collection instruments were sent by e-mail to the selected specialist nurses, containing: the characterization of the participants and the validation instrument containing the ND/NO and NI statements arranged according to the BHN.

The ND/NO and NI were assessed using a Likert scale with a score of 1 to 4, with the following definitions: 1. item not significant or not representative; 2. item needs major revision to be significant/representative; 3. item needs minor revision to be significant/representative; 4. item significant and representative. For items rated “2” or “3”, there was a field for suggestions. With regard to the ND/NO, the experts were asked if the statements were adequately classified according to the BHN; if the answer was no, they could objectively indicate which requirement the statement should be relocated to.

In order to update the set of diagnostic statements created, they were compared to the Systematized Nomenclature of Medicine International - Clinical Terms (SNOMED CT). It should be noted that in 2020, the CIE released SNOMED International and the partnership with ICNP^®^. However, SNOMED CT does not have a translation into Brazilian Portuguese, so it was necessary to have it analyzed by a licensed professional hired by the authors in order to analyze the linguistic equivalence of the statements prepared with those of SNOMED CT, and these statements are presented in the “Results” section.

### Data Analysis and Processing

The results were compiled in an Excel for Windows^®^ spreadsheet in order to formalize the database. The characterization data was analyzed using descriptive statistics (frequency and percentages), and the Content Validity Index (CVI) was used to measure the experts’ agreement regarding the validity of the statements. The CVI score was calculated considering the items evaluated with a score of 3 or 4, divided by the total sum of the responses to the items. Items with a CVI ≥ 0.80^(21)^ were validated. In addition to the statistical analysis, the experts’ suggestions for revising the statements, operational definitions and classification of human needs were analyzed and the relevant adjustments made.

### Ethical Aspects

This study was cleared by the Research Ethics Committee of the Pedro Ernesto University Hospital, in accordance with the principles established in Resolution 466/2012 of the National Health Council (CNS), under substantiated opinion no. 3.113.782, on January 16, 2019.

## RESULTS

Based on the terminology collected, 82 nursing diagnoses and 130 nursing outcomes were constructed, which were submitted to the process of cross-mapping with ICNP^®^ version 2019/2020 and subsequent analysis of equivalence and cardinality, which resulted in 60 nursing diagnoses and 34 constant nursing outcomes; and 22 nursing diagnoses and 96 nursing outcomes not included in ICNP^®^. A total of 556 nursing interventions were developed.

The nursing diagnoses, outcomes and interventions were subjected to content validation by specialist nurses. Of these, 56.7% were PhDs, with more than ten years’ training and professional experience and experience in hemodialysis of ten years or more. Meanwhile, 43.3% were nephrology specialists, with more than 5 years of training and professional experience and 5 years or more of experience in hemodialysis.

A total of 81 (98.8%) nursing diagnoses were validated along with their conceptual and operational definitions, 61 (74.5%) of which were allocated to the psychobiological needs group; 18 (21.9%) to psychosocial needs and 02 (2.4%) to psychospiritual needs, all with CVI ≥ 0.80. Only the nursing diagnosis/outcome “Adherence to Diet” was not validated. The statements are presented together with the code contained in the ICNP^®^ (Chart 1). When compared with SNOMED CT, 60 (73.2%) of the statements were in line with ICNP^®^.

**Chart 1:** Statements of nursing diagnoses/outcomes for people with chronic kidney disease on hemodialysis organized according to the Basic Human Needs Theory (CVI ≥ 0.80) – Rio de Janeiro, RJ, Brazil, 2024.

Psychobiological Needs (CVI ≥ 0.80)	
Oxygenation	Dyspnea (10029433)/Dyspnea, Absent (161938003); Hypoxia (10009608)/Hypoxia, Absent; Orthopnea (10013823)/Orthopnea, Absent;
Hydration	Azotemia/Azotemia, Improved/Azotemia, Absent; Electrolyte Imbalance (10033541)/Electrolyte Balance, Improved (10033518)/Electrolyte Balance, Adequate; Uremic Breath/Uremic Breath, Absent; Thirst, Increased/Thirst, Decreased/Thirst, Absent; Volume of Liquids, Increased/Volume of Liquids, Decreased/Volume of Liquids, Adequate/Volume of Liquids, Decreased/Volume of Liquids, Adequate; Xerostomia/Xerostomia, Improved/Xerostomia, Absent;
Nutrition	Underweight (10027316)/Body Weight, Improved/Body Weight, Adequate; Nutritional Intake, Impaired (10023009)/Nutritional Intake, Improved/Nutritional Intake, Adequate; Obesity (10013457)/Body Weight, Improved/Body Weight, Adequate; Risk of Overweight/Risk of Overweight, Decreased/Risk of Overweight, Absent; Overweight (10027300)/Body Weight, Improved/Body Weight, Adequate;
Elimination	Constipation (10000567)/Constipation, Improved/Constipation, Absent; Risk of Constipation (10015053)/Risk of Constipation, Decreased/Risk of Constipation, Absent; Vomiting (10025981)/Vomiting, Absent (10029181);
Sleep and rest	Insomnia (10010330)/Insomnia, Absent/Sleep, Adequate (10024930); Sleep, Inadequate/Sleep, Adequate (10024930);
Physical activity	Muscle Cramp (10046703)/Muscle Cramp, Improved/Muscle Cramp, Absent; Fatigue (10000695)/Fatigue, Decreased/Fatigue, Absent (10034727); Sarcopenia/Sarcopenia, Improved;
Sexuality and reproduction	Sexual Performance, Impaired (10001288)/Sexual Performance, Improved;
Physical and environmental safety	Risk of Allergy/Risk of Allergy, Decreased/Risk of Allergy, Absent; Risk of Fracture/Risk of Fracture, Decreased/Risk of Fracture, Absent; Risk of Infection (10015133)/Risk of Infection, Decreased/Risk of Infection, Absent; Risk of Fall (10015122)/Risk of Fall, Decreased/Risk of Fall, Absent;
Physical integrity	Gingivitis (10043312)/Gingivitis, Improved/Gingivitis, Absent; Skin Integrity, Impaired (10001290)/Skin Integrity, Improved (10028517); Pruritus (10010934)/Pruritus, Improved/Pruritus, Absent;
Vascular regulation	Pulmonary Congestion/Pulmonary Congestion, Improved/Pulmonary Congestion, Absent; Cardiac Output, Impaired (10025557)/Cardiac Output, Improved/Cardiac Output, Adequate; Peripheral Edema (10027482)/Peripheral Edema, Decreased/Peripheral Edema, Absent (10029020); Hemodialysis, Insufficient/Hemodialysis, Adequate; Hemorrhage at Vascular Access Site/Hemorrhage at Vascular Access Site, Decreased/Hemorrhage at Vascular Access Site, Absent; Hypertension (10009394)/Blood Pressure, within Normal Limits (10027647); Hypotension (10009534)/Blood Pressure, within Normal Limits (10027647); Risk of Chest Pain, Sudden/Risk of Chest Pain, Sudden, Decreased/Risk of Chest Pain, Sudden, Absent; Risk of Bleeding (10017268)/Risk of Bleeding, Decreased/Risk of Bleeding, Absent; Risk of Hypotension, Severe/Risk of Hypotension, Severe, Decreased/Risk of Hypotension, Severe, Absent; Risk of Vascular Injury/Risk of Vascular Injury, Decreased/Risk of Vascular Injury, Absent; Risk of Tissue Perfusion, Impaired/Risk of Impaired Tissue Perfusion, Decreased/Risk of Impaired Tissue Perfusion, Absent;
Thermal regulation	Fever (10041539)/Fever, Decreased/Fever, Absent;
Neurological regulation	Headache/Headache, Absent; Cognition, Impaired (10022321)/Cognition, Improved (10051540); Memory, Impaired (10001203)/Memory, Improved;
Hormonal regulation	Anemia, Chronic/Anemia, Chronic, Improved; Albumin, Decreased/Albumin, Improved/Albumin, within Normal Limits; Hyperglycemia (10027550)/Blood Glucose Level, within Normal Limits (10033685); Hyperlipidemia (10041055)/Hyperlipidemia, Improved/Hyperlipidemia, Absent; Hypoglycemia (10027566)/Blood Glucose Level, within Normal Limits (10033685); Hypovitaminosis (10009581)/Hypovitaminosis, Improved;
Sensory perception	Chills (10018045)/Chills, Absent; Pain, Chronic (10000546)/Pain, Chronic, Decreased/Pain, Chronic, Absent; Nausea (10000859)/Nausea, Absent (10028984);
Therapy and prevention	Dietary Regime Adherence (10030159)/Dietary Regime Adherence (10030159); Dietary Regime Non-Adherence (10022117)/Dietary Regime Adherence (10030159); Physical Exercise Regime Non-Adherence (10022657)/Physical Exercise Regime Adherence (10030163); Non-Adherence to the Fluid Regime (10022129)/Adherence to the Fluid Regime (10030171); Non-Adherence to the Medication Regime (10021682)/Adherence to the Medication Regime (10030192); Non-Adherence to Hemodialysis Treatment/Adherence to Hemodialysis Treatment;
Gregaria	Family Process, Impaired (10023078)/Family Process, Improved/Family Process, Adequate; Relationship, Ineffective/Relationship, Improved/Relationship, Effective; Socialization, Impaired (10001022)/Socialization, Improved/Socialization, Adequate;
Recreation and leisure	Leisure, Harmed/Leisure, Improved;
Emotional security	Anxiety (10000477)/Anxiety, Decreased/Anxiety, Absent; Comfort, Impaired/Comfort, Improved/Comfort, Increased; Impotence (10001578)/Impotence, Improved/Impotence, Decreased (10027120); Fear (10000703)/Fear, Decreased/Fear, Absent; Anger (10045578)/Anger, Decreased/Anger, Absent; Sadness, Chronic (10000551)/Sadness, Chronic, Improved;
Self-esteem, self-confidence and self-respect	Self-esteem, Negative/Self-esteem, Positive;
Freedom and participation	Coping, Ineffective/Coping, Improved/Coping, Adequate; Denial of Illness/Acceptance of Illness; Denial of Treatment/Acceptance of Treatment; Resilience, Impaired/Resilience, Improved/Resilience, Increased;
Health education and learning	Self-care, Impaired/Self-care, Improved/Self-care, Adequate; Health Literacy, Impaired/Health Literacy, Improved/Health Literacy, Adequate;
Self-realization	Labor Capacity, Decreased/Labor Capacity, Improved/Labor Capacity, Increased;
Religiosity and spirituality	Spiritual Suffering/Spiritual Suffering, Decreased/Spiritual Suffering, Absent; Religious Bonding, Impaired/Religious Bonding, Improved;

The experts suggested gradations in the nursing outcomes, which resulted in 242 validated nursing outcomes, of which 182 were allocated to the psychobiological needs group; 54 to psychosocial needs and 06 to psychospiritual needs, all with CVI ≥ 0.80 (Chart 1). When compared to SNOMED CT, 44 (33.8%) of the statements were in line with ICNP^®^.

At the same time, 533 (95.8%) nursing interventions were validated, of which 366 (65.8%) were allocated to the psychobiological needs group; 152 (27.3%) to psychosocial needs and 15 (2.7%) to psychospiritual needs, all with CVI ≥ 0.80 (Chart 1). Of these, 23 (4.1%) nursing interventions were not validated, which are: “Monitor and advise on the importance of water restriction”; “Instruct the patient on the amount and types of food suitable for ingestion”; “Calculate the patient’s BMI”; “Advise the patient and family on the importance of food restriction for their health condition”; “Advise the patient to eat fractional meals every three hours”; “Assess the patient’s condition for physical exercise”; “Advise the patient on the importance of low-intensity exercise to increase tolerance to activity”; “Take blood pressure at regular intervals, preferably every 1 hour or 30 minutes or less frequently depending on the case throughout the hemodialysis session”; “Record body temperature”; “Monitor vital signs”; “Check that blood glucose levels are satisfactory after insulin administration”; “Assess the patient’s adherence to the diet and exercise regime”; “Encourage the patient to take up physical activity”; “Assess the effectiveness of pain control measures”; “Encourage the patient to take part in leisure activities”; “Help the patient to identify anxiety-provoking situations”; “Teaching activities that reduce anxiety”; “Encouraging the patient to perform regular physical activities, according to their ability”; “Providing comfort to the patient”; “Encouraging the patient’s self-confidence”; “Promoting the patient’s self-esteem”; “Assessing the patient’s ability to learn”; “Explaining to the patient the importance of self-care for their independence”.

Due to the large number of statements of interventions, these were presented according to the number created and validated, according to the BHN (Table 1).

The ICNP^®^ Terminology Subset for people with chronic kidney disease on hemodialysis was registered with the Brazilian Book Chamber (CBL).

**Table 1:** Nursing intervention statements for people with chronic kidney disease on hemodialysis organized according to the Basic Human Needs Theory (CVI ≥ 0.80) - Rio de Janeiro, RJ, Brazil, 2024.

Basic Human Needs	Interventions created (N)	Interventions validated (N)
Oxygenation	14	14
Hydration	25	23
Nutrition	18	15
Elimination	19	19
Sleep and rest	13	13
Physical activity	21	19
Sexuality and reproduction	12	12
Physical and environmental safety	34	34
Physical integrity	27	27
Vascular regulation	71	70
Thermal regulation	11	10
Neurological regulation	19	18
Hormonal regulation	33	30
Sensory perception	29	28
Therapy and prevention	34	34
Gregarious	24	24
Recreation and leisure	09	08
Emotional security	42	38
Self-esteem, self-confidence and self-respect	16	14
Freedom and participation	30	30
Health education and learning	29	27
Self-realization	11	11
Religiosity and spirituality	15	15
**Total**	556	533

## DISCUSSION

The Terminological Subset of the ICNP^®^ for people with chronic kidney disease on hemodialysis is considered an important reference guide for nursing practice, which aims to help nurses during their clinical practice to promote systematized care for this clientele, using evidence-based practice; direct clinical reasoning, decision-making and provide support to nurses during the execution and recording of the Nursing Process.

This study showed that most of the nursing diagnosis/outcome statements constructed were validated by the specialists, and were included in the ICNP^®^. This data shows that the large number of pre-coordinated concepts contained in ICNP^®^ version 2019/2020 expresses the use of standardized languages in professional nursing practice and ensures the reliability of ICNP^®^ as a technological tool for insertion into information systems, recording care and identifying sensitive indicators of nursing practice worldwide, with a view to the scientific and technological development of the profession^(22)^.

The psychobiological needs encompassed a large number of statements of nursing diagnoses, outcomes and interventions, the most common of which is affected in people with chronic kidney disease, and the pre-coordinated concepts found in this group reflect the nursing phenomena identified in their clinical condition as a result of the numerous biological repercussions of the disease and treatment on the individual’s body. In this way, nurses play a fundamental role in the care process, as they can promote effective treatment and good impacts on personal needs.

With regard to the Need for Vascular Regulation, “Insufficient Hemodialysis” and “Hemorrhage at Vascular Access Site” stand out as diagnoses that have been highly validated by specialists and are not included in the ICNP^®^. To this end, nurses should pay attention to the Kt/V index, which assesses the dose of dialysis offered to the individual. Thus, when it is below 1.2 it means that there is a decrease in the efficiency of the hemodialysis received by the individual. Consequently, there will be a reduction in the removal of nitrogenous slags and liquids, and increased vulnerability to developing water overload^(23)^.

Another relevant aspect for nurses practicing hemodialysis is vascular access, whether it’s a double lumen catheter (DLC) or an arteriovenous fistula (AVF). The AVF is a long-term access with a low risk of infection and a low incidence of mortality. However, thrombosis is one of the major complications that causes the loss of this vascular access, and its occurrence is usually preceded by hypotension, narrowing of the vessel, reduced blood flow, repeated punctures in the same place, bruising and bleeding. In this sense, the nurse must be aware that its functioning and durability depend on the care of both the person and the healthcare team. When caring for people with CKD, nurses are responsible for monitoring possible signs of vascular access complications. Their duties range from guiding the individual through washing the arm of the AVF to carrying out an accurate physical examination^(24)^.

On the other hand, in Therapeutic and Prevention Needs, it should be highlighted the diagnosis “Non-adherence to Hemodialysis Treatment”, which is not included in the ICNP^®^ and which may be related to the social determinants of this population. A study carried out in the north-east of Brazil with 79 people on chronic hemodialysis revealed that most of them had reduced their attendance at HD sessions with a consequent reduction in the dose of dialysis, possibly due to the rigidity of HD schedules combined with the time spent in sessions and the occurrence of symptoms after treatment, with slow recovery^(25)^. However, it was observed that there is a lack of studies on the access and adherence of people with CKD to hemodialysis therapy, which emphasizes the importance of validating this diagnosis. As a result, nurses in their clinical practice can routinely assess this data, since adherence is a dynamic behavioral habit and is directly linked to quality of life.

In people affected by CKD, the need for hydration is a critical component of hemodialysis treatment. Studies show that excessive consumption of fluids and sodium are the main factors related to water overload in these people. In addition, this clientele has difficulty adhering to water and dietary restrictions^(23)^. In this sense, the nursing team plays a very important role in the early detection of possible complications and changes in the individual’s general condition caused by the disease and treatment. It is therefore necessary for nurses to develop educational and motivational measures that promote optimal interdialytic weight maintenance.

As for psychosocial needs, the Needs for Gregariousness and Emotional Security were represented by some diagnoses, such as: “Impaired socialization” and “Anger”, which refer to the social and emotional reality experienced by people with chronic kidney disease and their families. In this way, the new living conditions imposed on people undergoing hemodialysis make it difficult for them to accept their new physical state, laden with the marks of the hemodialysis process. Faced with these needs, a social, family and multi-professional support network during this phase is crucial, as the person needs someone available to share all the impacting feelings that will arise as a result of the treatment^(26, 27)^. Therefore, nurses in their practice should value the relationship between the individual and the caregiver, who should be given opportunities and their needs pointed out, thus sharing the responsibility for care.

Another aspect worth highlighting is health literacy. This aspect can be corroborated in a study which showed that 80.9% of chronic renal failure patients undergoing hemodialysis had inadequate health literacy. Thus, nurses need to be aware of this fact, as this ED can contribute to worse health outcomes for people with chronic kidney disease due to its influence on the mechanisms of knowledge, attitude and behavior, since these people are unable to process the health information they receive and transform it into sufficient knowledge to manage their pathology^(28)^. In this way, nurses in their practice can measure the lack of knowledge and level of self-management of CKD in their care plan, helping to avoid hospitalizations, poor adherence to treatment and cardiovascular events.

Finally, we have included Psychospiritual Needs, which allocated 02 NDs to the religiosity and spirituality subgroup: “Spiritual Suffering” and “Impaired Religious Bond”. Although the literature provides a lot of evidence for the formulation of these diagnoses, it can be seen that their approach is still incipient in nephrology studies. Nursing diagnoses related to religiosity and spirituality are not very specific. Spirituality is understood as the individual search for meaning and purpose in life through the transcendence of the self. Religiosity, on the other hand, implies the human being’s relationship with a transcendent being, i.e. the extent to which the individual believes in, follows and practices a religion^(29, 30)^.

In this context, it is important to emphasize the need to properly incorporate this theme into nurses’ care practices. With this, fruitful results can contribute to situational coping, strengthening, social support and confrontation of pain and improvement in the perception of quality of life and renal clinical status.

As for the statements of nursing interventions, those not validated by the experts were represented by only 4.1%, which is considered a low percentage. This may be related to a methodological bias in this study, which refers to the impossibility of the experts to evaluate the nursing interventions separately, since they were evaluated in groups for each nursing diagnosis. In any case, the validated statements can be included in a care plan to contribute to an assessment focused on the main needs affected, facilitating the identification of favorable results with the team.

In terms of contribution, the statements of nursing diagnoses, outcomes and interventions validated in this study and considered not to be included in the ICNP^®^ could be suggested for inclusion in the classification, with the aim of filling the gaps in this system and promoting continuity in the terminology’s life cycle. With this, this subset aims to standardize language, improving patient safety through clinical indicators of practice and for practice, thus developing nursing as a science. In addition, it has been cross-mapped with SNOMED CT, thus demonstrating an advance in Brazilian studies, since it still lacks translation, interoperability with ICNP^®^ and a review of the method.

This subset is a technological and educational product that helps nurses to develop and record the essential elements of their professional practice, stimulate their critical thinking, contribute to recognizing the role of nurses in health care and improve the quality of nursing care. Although the target audience is nursing assistants, this material can be used in undergraduate and postgraduate teaching environments (theoretical and/or practical classes and curricular internships), in research and in nursing management. Finally, it should be noted that during their clinical practice, nurses will be able to identify nursing phenomena that have not been covered in this subset, and can make the changes they deem necessary during the care they provide.

A limitation of the study is that clinical validation was not carried out, which is an important step in consolidating the use of ICNP^®^ by nephrology nurses, in order to integrate the knowledge produced in academia with clinical practice. However, the authors recognize the need for it in future research.

## CONCLUSION

The research made it possible to construct and validate the terminological subset of the ICNP^®^ for people with chronic kidney disease on hemodialysis, in the light of the Basic Human Needs Theory, meeting the proposed objective. Initially, 82 nursing diagnoses, 130 nursing outcomes and 556 nursing interventions were constructed. Subsequently, the content of the statements was validated by specialist nurses, resulting in 81 statements of nursing diagnoses, 242 statements of nursing outcomes and 533 statements of nursing interventions, with a predominance of statements related to psychobiological needs.

It is hoped that the proposal of this subset will contribute to the standardization of the language used in nursing diagnoses, results and interventions, in the elaboration and recording of these essential elements of nursing practice, in the improvement of communication, clinical reasoning, decision-making and in the consolidation of the nurse’s work process, with the aim of improving the quality of nursing care.

Finally, it should be emphasized that this is the first terminological subset for the care of people with chronic kidney conditions undergoing hemodialysis treatment, representing a starting point for future studies such as the validation of clinical applicability.

## References

[1] Jesus NM, Souza GF, Rodrigues CM, Almeida No OP, Rodrigues DDM, Cunha CM (2019). Quality of life of individuals with chronic kidney disease on dialysis. J Bras Nefrol.

[2] Brasil (2023). Dia Mundial do Rim: ANS destaca importância da prevenção da doença renal crônica [Internet].

[3] Sociedade Brasileira de Nefrologia (2023). Censo 2023 [Internet].

[4] Hagemann PMS, Martin LC, Neme CMB (2019). The effect of music therapy in hemodialysis patients. Braz J Nephrol.

[5] Menezes HF, Camacho ACLF, Nóbrega MML, Fuly PSC, Fernandes SF, Silva RAR (2020). Paths taken by Brazilian Nursing for the development of terminological subsets. Rev Latino-Am Enfermagem.

[6] Brasil (2024). Dispõe sobre a implementação do Processo de Enfermagem em todo contexto socioambiental onde ocorre o cuidado de enfermagem.

[7] Horta WA (2018). Processo de Enfermagem.

[8] Garcia TR (2020). Classificação Internacional para a prática de Enfermagem (CIPE^®^): versão 2019/2020.

[9] Cubas MR, Nóbrega MML (2015). Atenção primária em saúde: diagnósticos, resultados e intervenções de enfermagem.

[10] Carvalho CMG, Cubas MR, Nóbrega MML (2017). Brazilian method for the development terminological subsets of ICNP^®^: limits and potentialities. Rev Bras Enferm.

[11] Clares JWB, Guedes MVC, Freitas MC (2020). International Classification for Nursing Practice in Brazilian dissertations and theses. Rev Eletr Enferm.

[12] Lins SMSB, Espírito Santo FH, Fuly PSC, Garcia TR (2013). Subset of ICNP^®^ diagnostic concepts for patients with chronic kidney disease. Rev Bras Enferm.

[13] Menezes HF, Camacho AC, Sant’Anna RM, Matos TL, Santos IS, Silva AB (2023). ICNP^®^ terminology subset for people with chronic kidney disease under conservative treatment. Acta Paul Enferm.

[14] International Council of Nurses (2018). Guidelines for ICNP^®^ catalogue development [Internet].

[15] Santos JO (2022). Subconjunto Terminológico da CIPE^®^ para pessoas com doença renal crônica em hemodiálise [dissertação].

[16] Santos JO, Lins SMSB, Nóbrega MML, Tavares JMAB, Menezes HF, Silva HCDA (2023). Specialized nursing terminology for chronic kidney patients on hemodialysis. Esc Anna Nery.

[17] International Organization for Standardization (2023). ISO 18104: Health informatics — Categorial structures for representation of nursing practice in terminological systems.

[18] Menezes HF, Camacho ACLF, Monteiro PP, Santos IS, Pereira AB, Prado NCC (2024). Clinical validation of the terminological subset for people with chronic kidney disease undergoing conservative treatment. Rev Esc Enferm USP.

[19] Vidigal PD, Garcia TR, Santos ML, Camacho ACLF, Souto MD, Borges GG (2018). ICNP^®^ terminology subset for patients with cancer-associated venous thromboembolism. Acta Paul Enferm.

[20] International Organization for Standardization (2016). ISO 12300: health informatics: principles of mapping between terminological systems.

[21] Rocha CCT, Lima DM, Menezes HF, Silva RS, Sousa PAF, Silva RAR (2022). Nursing diagnoses for people living with hiv: relationships between terminologies. Texto Contexto Enferm.

[22] Clares JWB, Fernandes BKC, Guedes MVC, Freitas MC (2019). Specialized nursing terminology for the care of people with spinal cord injury. Rev Esc Enferm USP.

[23] Fernandes MICD, Carino ACC, Gomes CST, Dantas JR, Lopes MVO, Lira ALBC (2021). Content analysis of the diagnostic proposition risk of excessive fluid volume in hemodialysis patients. Rev Esc Enferm USP.

[24] Silva EF, Lins SMSB, Tavares JMAB, Marta CB, Fuly PSC, Broca PV (2020). Nursing care with surgical arteriovenous shunt in renal dialysis: a validation study. Rev Bras Enferm.

[25] Dantas LG, Rocha MS, Cruz CMS (2020). Non-adherence to hemodialysis, perception of the illness, and severity of advanced nephropathy. J Bras Nefrol.

[26] Santos GLC, Alves TF, Quadros DCR, Giorgi MDM, Paula DM (2020). The person’s perception about its condition as a chronic renal patient in hemodialysis. Rev Fun Care Online.

[27] Rodrigues KA, Silva EM, Barbosa LDCS (2020). Biopsychosocial repercussions in patients submitted to hemodialytic treatment. Res Soc Dev.

[28] Bezerra JNM, Lessa SROÓ MF, Luz GOA, Borba AKOT (2019). Health literacy of individuals undergoing dialysis therapy. Texto Contexto Enferm.

[29] Leimig MBC, Lira RT, Peres FB, Ferreira AGC, Falbo AR (2018). Quality of life, spirituality, religiosity, and hope in chronic renal disease patients in hemodialysis. Rev Soc Bras Clin Med.

[30] Ferreira GSM, Fernandes PFCCB, Oliveira LC, Pinto JR, Ferreira IBM, Gurgel FF (2022). Religiosity, spirituality and quality of life in patients with chronic kidney disease who underwent hemodialysis in northeastern Brazil. Res Soc Dev.

